# The Role of Front-of-Package Nutrition Labels with and without Explanatory Videos on Parent and Child Food Choices

**DOI:** 10.3390/nu15184082

**Published:** 2023-09-21

**Authors:** Dan J. Graham, Rachel G. Lucas-Thompson, Gina Slejko

**Affiliations:** 1 Department of Psychology, College of Natural Sciences, Colorado State University, Fort Collins, CO 80523, USA; 2 Department of Human Development and Family Studies, College of Health and Human Sciences, Colorado State University, Fort Collins, CO 80523, USA; lucas-thompson.rachel.graham@colostate.edu; 3 Department of Marketing, College of Business, Colorado State University, Fort Collins, CO 80523, USA; gina.slejko@colostate.edu

**Keywords:** nutrition labeling, front-of-package labels, food choice, education campaign

## Abstract

The aim of this research was to determine whether parent/child pairs choosing products from a grocery aisle labeled with front-of-package (FOP) nutrition labels would make more healthful choices than pairs who viewed the same items without labels, and to determine the added value of viewing an explanatory video before choosing. In this experiment, 175 parent/child pairs chose USD 20 worth of packaged foods and beverages from a grocery aisle in a research laboratory and were randomly assigned to see products that either did or did not have 0–4-star FOP labels, with more stars indicating more healthful products. Among those participants with access to FOP labels, half were randomly assigned to view a 30 s video explaining the FOP labels before selecting foods. Participants who saw the explanatory video before selecting among products with FOP labels chose foods with significantly more stars than participants who saw the FOP-labeled products without the video; however, there was no significant difference in mean stars on selected products between the group that saw the videos and the control group that saw neither the video nor FOP labels. We conclude that explaining new FOP labels to consumers may be necessary for the labels to prompt more healthful choices.

## 1. Introduction

Given the public health crisis resulting from poor nutritional quality of consumer diets, policy makers and public health experts are continually seeking new strategies to improve consumer health [1]. The use of front-of-package (FOP) nutrition labeling is one strategy that has received growing attention from researchers and policy makers and has been recognized as an important policy issue by the World Health Organization [2] and the Organization for Economic Co-operation and Development [3]. Consequently, some countries, such as Australia, New Zealand, France, and Chile have adopted national FOP nutrition labels, and others, such as the United States of America and the Netherlands are considering doing so [4].

The debate over whether the U.S. should adopt an FOP nutrition label policy is making news as special interest groups continue to put pressure on the FDA to mandate this practice [5]. Proponents note that eating diets lower in certain nutrients (e.g., saturated/trans fat, sodium, and added sugars) benefits health in a variety of ways, including a lower incidence of cardiovascular disease, diabetes, and obesity [6,7,8,9], so if FOP labeling interventions serve as an effective nudge to choose foods lower in these nutrients, this has the potential to confer significant public health benefit. Indeed, following the September 2022 White House Conference on Hunger, Nutrition, and Health, Senator Cory Booker told National Public Radio that one key priority to promoting healthier eating in the U.S. that came out of this conference was “front-of-package labeling with critical information for consumers [so] that consumers will make better choices” [10].

FOP labels under consideration can be classified as either reductive or interpretive. Whereas reductive labels (e.g., Reference Intakes) only summarize the quantity and percent daily allowance in a single serving of food, interpretive labels provide additional guidance regarding the healthfulness of the food. Interpretive labels are further differentiated with the presence of nutrient-specific information (e.g., Multiple Traffic Lights (MTL) and warning labels), the provision of a summary indicator that presents the overall nutritional composition of the food (e.g., Nutri-Score), and hybrid labels that include both types of information (e.g., Health Star Rating [HSR]). Summary indicators such as Nutri-Score, which was adopted in France in 2017, have been shown to be more effective than nutrient-specific labels as indicated with more healthful food choices among consumers [11,12]. Although some labeling systems have demonstrated greater effectiveness than others, it is important to note that all systems have limitations, and that labeling individual foods and beverages as healthy or unhealthy can obscure the fact that foods are not consumed in isolation, but rather as part of a diet. It is the entirety of the diet that is best evaluated for its relative healthfulness [13,14], and labels on individual foods can be used to help identify component parts of an overall healthy diet, but it is still possible (e.g., by consuming a very limited number of products) to eat foods that have all been labeled as healthy without consuming an overall healthy, balanced diet.

Research examining the primary FOP labels used in the EU has revealed that interpretive labels with summary indicators are most effective at increasing the overall nutritional quality of a shopping cart of products (e.g., Ducrot et al. [15]). Label formats examined in the Ducrot et al. study [15] included two nutrient-specific labels, (1) Guideline Daily Amounts (GDA) and (2) Multiple Traffic Lights (MLT), and two summary labels, (1) 5-Color Nutritional Label (5-CNL) and (2) Green Tick (Tick). In their analysis, the authors found that the 5-CNL performed well consistently, across socioeconomic measures, in promoting healthier food choices [15].

Compelling evidence points to the use of summary indicators as an effective tool to aid in healthful food choice, and nutrient-specific warning labels (i.e., “high in sodium”), have been shown to be more effective than reductive labels (i.e., Reference Intakes) in prompting more healthful food choices and have therefore been adopted in Chile [16]. Countries such as Brazil, Uruguay, and Canada, and several U.S. states and cities, have therefore also explored the use of these labels to signal foods high in certain nutrients, particularly sugar (see [17]).

Overall, existing research demonstrates that FOP labels across formats are effective at raising consumer awareness around product healthfulness [18,19], but findings have been mixed regarding purchase behavior [19]. Thus, available data used to guide policy recommendations suggest that different types of FOP labels, among different populations, have varying effects on consumer behavior. Talati et al. [12] examined the effect of five labels on choice across 12 countries. The authors found that while all five formats had a positive influence on the nutritional quality of product choice, compared to conditions in which there was no FOP label present, Nutri-Score and MTL produced the strongest results. The HSR, which has been advocated for in the United States, showed a weaker level of improvement compared to the Nutri-Score and MTL formats.

Taken together, the efficacy of FOP labels is highly contextual. Therefore, acknowledgement of the effects of culture, socioeconomic status, and health literacy on FOP label impact has been the focus of some concern [20]. Recent research in this area has focused on country-specific outcomes with the aim of developing FOP labeling strategies that address culture- and country-specific needs. For example, Santos et al. (2020) demonstrate that a Traffic Light (TL) label was most preferred by adult Portuguese consumers and this preference was matched with higher levels of label comprehension (i.e., correctly identifying more nutritious options), compared to Guideline Daily Amounts, Nutri-Score, and Health Star Rating [21]. Taking a different approach, Todd et al. (2022) studied the comprehension of various FOP label formats among South African adults while also leveraging qualitative research methods to develop themes for label design improvements [22]. Findings revealed that simpler and clearer labels may have a positive impact on healthy food choice. Corroborating these findings were those from a recent study by Pettigrew et al. [23], who included New Zealand, India, and China in their analysis of FOP formats, to assess the role of color and simplicity of the HSR format in comprehension and purchase intention. Their findings show that, overall, the simpler formats (i.e., those with only summary indicators) performed better and, interestingly, India stood apart with Indian consumers showing greater comprehension for a full-color hybrid (i.e., summary indicator paired with nutrient-specific information) relative to the monochrome and simplified full-color formats [23].

The current research also takes a country-specific and education-based perspective on the examination of FOP label efficacy. In particular, the U.S. Institute of Medicine (now the National Academy of Medicine) recommended an interpretive nutrient-specific summary indicator label [24] but no FOP labels have been adopted at the national level. In the proposed design, a simple visual designation is created to indicate whether a product contains a healthful amount of three key nutrients to limit: saturated/trans fats, sodium, and added sugars. Labels using this recommended approach have been tested in several different settings (e.g., [25,26]), with some data indicating that FOP labels designed with these guidelines can be difficult for consumers to interpret (e.g., Graham and Mohr [27]). However, Graham and Mohr [27] studied how these labels were used online, rather than in a brick-and-mortar setting, and asked about healthfulness ratings, rather than examining actual food choices. It is important to understand if FOP labels designed in accordance with the IOM recommendations influence not only healthfulness perceptions and hypothetical food choices, but actual food choices among consumers who will receive the selected foods to take home with them, when shopping for groceries in real life.

Overall, FOP labels positively affect consumers’ abilities to discern healthfulness among commonly purchased foods. However, it is important to note that variance exists in the extent to which consumers attend to and use FOP labels in making food choices [28]. Importantly, while demographic and socioeconomic factors are not significant predictors of label use among some populations (e.g., [29]), nutritional knowledge does positively affect label use and comprehension [28,30,31]. Therefore, one strategy for leveraging the effectiveness of policies requiring FOP labeling is the development of tools aimed at educating consumers around the importance, meaning, and use of FOP labels.

While educational tools have been found to be helpful in increasing use and understanding of Nutrition Facts panels [32], little research has examined the efficacy of educational tools on the comprehension and use of FOP nutritional labels specifically. An exception is work by Graham et al. [33] who investigated the use of point-of-purchase FOP educational signage on child and parent food choices. Their findings point to a positive impact of in-aisle signage on food choices. Specifically, participants in this study with access to FOP labels and explanations of these labels chose products lower in saturated fat compared to when no explanatory signage was present. Although a review of the effects of educational tools on nutritional label use and understanding concluded that educational tools have a positive effect on these outcomes [32], the 17 studies reviewed were educating consumers about use of Nutrition Facts panels, rather than FOP labels. It is possible that educational efforts are less necessary for consumers to understand and use FOP labels that convey information in a relatively simplified manner, such as interpretive nutrient-specific summary indicator labels. Therefore, the current research seeks to advance this area of knowledge by testing the effect of an ecologically valid educational tool and interpretive nutrient-specific summary indicator FOP labels on food choice.

Finally, responding to the call for more information about the role of children in food decision making [34], this research was conducted with parent–child pairs. Currently, there is little experimental research on nutrition labeling and food choices that includes children (e.g., [16,35,36]. For instance, in a recent review of the effect of FOP labeling on food choice, there is no delineation among respondents based on age [37], because most research has studied only adult participants, although certain types of FOP labels (i.e., those that use interpretive symbols, such as stars or colors) would be more readily interpretable than others (i.e., those that use text) among children, particularly children who cannot yet read. Indeed, in South Korea, the color-coded traffic light FOP labeling system applies only to products that are primarily marketed toward children [38].

The present study examines the effects of interpretive nutrient-specific summary indicator FOP labels and explanatory videos on food choices among parent/child pairs. Pairs were randomly assigned to one of three conditions: 1. Control—no labels and no video; 2. FOP labels only (FOP Only)—all products these participants saw were labeled with 1–4-star FOP labels based on the IOM’s recommendations and the results of Graham and Mohr [27]; and 3. FOP labels and video (FOP + Video)—participants randomly assigned to this condition viewed a brief (30 s) public-service announcement video describing the FOP labels and their meaning before beginning the food selection task. This video was embedded in a series of eight brief videos viewed in a separate building, ostensibly as part of a different study on parent/child reactions to various advertisements.

It was hypothesized that

FOP labels alone (FOP Only) would not produce significantly different food choices compared with no labels (Control group).
The FOP + Video group would choose products that had, on average, more stars on their FOP labels relative to the FOP Only group and the no-label Control group.

## 2. Materials and Methods

Participants in this randomized controlled trial were 175 pairs of one parent/caregiver and one 5–10-year-old child who were recruited as part of a larger study that also examined parenting style. Children ranged in age from 5 to 10 years (*M* age = 7.66 years, *SD* = 1.32; 47% female). Parents/caregivers (*M* age = 38.40 years, *SD* = 6.85) were mostly female (89%). The majority of caregivers (76%, *n* = 133) spoke English during the visit, but 24% (*n* = 42) spoke Spanish; in terms of race/ethnicity, the majority self-identified as White (86%, *n* = 150), but others identified as Black (2%, *n* = 4) or chose not to report their race/ethnicity (12%, *n* = 21). In addition, the majority of caregivers were married (69%, *n* = 121) or not married but living with their significant other (6%, *n* = 10). On average, dyads belonged to families with a combined family income of USD 50,000–75,000 a year, and on average, parents had obtained a college degree. However, parental education ranged: 9% did not complete high school, 6% completed high school, 2% received vocational training beyond high school, 15% completed some college, 33% completed a college degree, and 35% received graduate or professional education. Recruitment was conducted via email solicitation, social media advertisements, in-person community events, and in partnership with a community organization that serves predominately Latinx families. Pairs who spoke English or Spanish were recruited to participate in 2 studies, conducted back-to-back and lasting approximately 90 min in total. To reduce the influence of demand characteristics on parent and child behavior, and thereby increase the likelihood that any differences in food choice by condition were driven by exposure to the explanatory video and FOP labels rather than participant expectations, participants responded to a recruitment advertising two separate studies to be completed together. Study 1 was billed as a marketing study seeking feedback on a variety of TV advertisements. Study 2 was described as a grocery shopping task.

Adult participants were excluded from participating if they did not speak English or Spanish and did not have a child aged 5 to 10 years old. Participants were recruited through (i) flyers in schools, community clinics, hospitals, physicians’ offices, and other local facilities, (ii) letters to area families with school-aged children, (iii) informational sessions at community events and churches, (iv) newspaper, radio, and public service announcements, (v) e-mails to school and other community listservs with parents of school-aged children; and (vi) a community health promotion organization that primarily serves low-income and Hispanic families. Materials invited families to participate in two studies to better understand parent and child impressions of product advertisements and parent–child interactions while grocery shopping. Participants came to an on-campus laboratory to complete Study 1 (video rating) and moved to a separate lab in a nearby building on campus for Study 2 (food selection task in mock grocery/convenience store aisle). Both studies are described in detail below:

Study 1: Video Rating. After obtaining parent consent and child assent, parents and children participated in what was described as a marketing study. Parents and children viewed a series of 7 advertisements. Dyads in the FOP Only condition and the Control group viewed a series of 7 unfamiliar product ads (taken from other English-speaking countries). All ads were appropriate for children and included Spanish subtitles. Dyads in the FOP + Video condition (i.e., those participants randomly assigned to see both FOP labels during the shopping task and an explanatory video) viewed the same ads, but also viewed an FOP-label public service announcement (PSA)-style educational video created by the study team. This educational advertisement explained the purpose of the relevant “Healthy Stars” FOP labels and explained how to use these labels to evaluate the healthfulness of foods. This education was modeled after a PSA/commercial advertisement and was 30 s long. After viewing each ad, participants were asked two questions requiring them to evaluate the quality of the ad as well as the main message of the ad: (1) “Can you tell me how much you disliked vs. liked the commercial you just saw?” from “liked a lot” to “disliked a lot” (the children’s version of the questionnaire also had corresponding emoji-style face images showing a very happy face to a very unhappy face), and (2) the open-ended question “Please describe what this commercial was about.”

**Figure 1:**
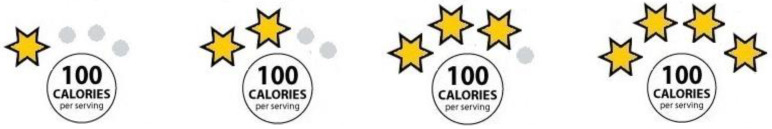
Sample FOP labels.

Study 2: Food Selection. In a different laboratory in a nearby university building, two mock grocery aisles were set up on opposite walls, stocked with 90 common foods and beverages including canned fruits, vegetables and legumes, pastas and pasta sauce, cookies, peanuts, crackers, soups, cereal bars, peanut butter, jelly, breakfast cereals, snack foods such as pretzels and potato chips, juice, soda, canned fish and meats, and dried fruit. The products on the shelves were in the same locations in each aisle, with the only difference being the presence of added 1–4-star FOP labels in the upper right-hand corner of all items included in one of the aisles (see Figure 1 for example label); no FOP labels were added to the products in the other aisle. During each visit, the aisle that was not being used was covered with a large tarp.

After obtaining parent consent and child assent to participate in Study 2, participants were provided with a grocery basket and instructed that they could choose up to USD 20 worth of products from the grocery aisle to take home with them and they were further instructed that parents and children should work together to make their choices. The specific prompt provided was “We’ll give you a $20 budget and ask you to pick out foods like you were grocery shopping together, and you’ll get to take these foods home today. You can choose any foods you want, but you can’t go over the $20 budget, and you won’t get to keep any money left over if you spend less than $20.” No instructions were provided regarding the FOP labels. All prices were rounded to the nearest whole dollar amount to facilitate ease of calculating USD 20 worth of products for participants. Once participants had selected their preferred products, they notified the experimenter that they were finished. The experimenter then calculated the total price of the items in the grocery basket, and if the price exceeded USD 20, participants were asked to return something in order to get to USD 20 or less. At the conclusion of this task, the experimenter made note of which products were selected and the parent completed a brief food purchasing questionnaire asking how frequently they buy the foods that they selected in this Study 2 food selection task. Finally, parents completed questionnaires for approximately 30 min while children played with toys provided by the experimenter.

**Figure 2:**
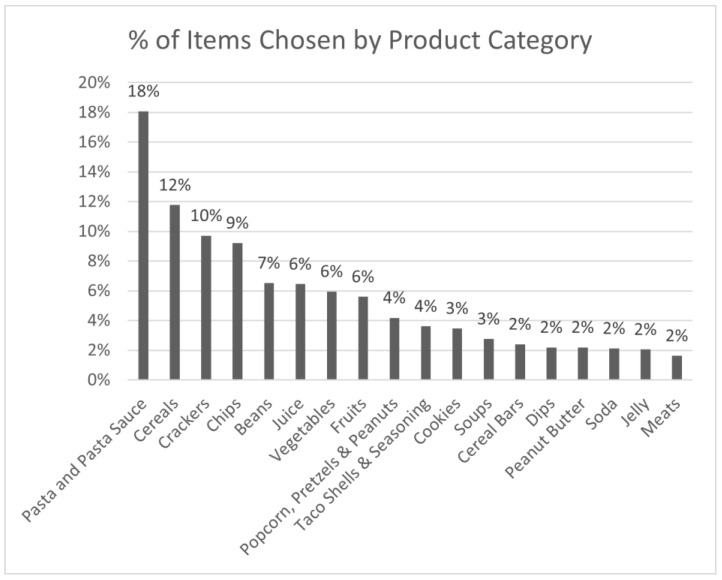
Percent of products purchased by category.

To delay directing attention to the health/nutrition focus of the study, parents responded to health-related questions after completing the food selection task. Parents were permitted to skip any questions that they were not comfortable answering. The questionnaire assessed parent/child overall health (i.e., “Rate your [your child’s] overall health.”), parent perception of the importance of health and healthy eating (e.g., “How important is it to you to purchase healthy food?”), perceptions of what makes some products healthy (e.g., “How important is sodium in determining whether a food is healthy?”), and awareness of federal nutrition information programs and use of food labels (e.g., “How often do you read the nutrition information on food labels before purchasing foods or beverages?”); (questions were taken from the National Health and Nutrition Examination Survey.) Parents completed a brief battery of measures to assess potential control/confounding variables, including demographics (e.g., age, race, ethnicity, marital status, household income, and education), home environment (e.g., “There is very little commotion in our home”), family resources (e.g., “[We have] money to buy necessities”), family well-being (e.g., purchasing behavior/intention for a selected list of products (i.e., those categories of foods and beverages available in the study aisle—see Figure 2)), how much the participating child likes these products, parenting style in general (e.g., “I give praise when my child is good.”) and in relation to eating (e.g., “I do not allow my children to eat junk foods.”), and dietary behaviors for the previous 30 days using the Five Factor Screener from the 2005 NHIS Cancer Control Supplement. In addition, parent and child height and weight were measured via a stadiometer/calibrated scale.

After completing these procedures for Study 1 and Study 2, dyads were debriefed about the aims of the research and the link between the two studies. As they departed, dyads received the foods and beverages they selected from the grocery aisle, a USD 10 gift card for their participation in Study 1, and a USD 20 gift card for their participation in Study 2.

## 3. Results

The criteria for assigning stars to products were delineated by the Institute of Medicine [24] and were guided using the quantities of added sugars, sodium, and saturated/trans fats contained in the products. Of the 90 available products, 18 products received 4 stars, 32 products received 3 stars, 13 received 2 stars, and 27 received 1 star. Products ranged in price from USD 1 to 5 (mean of USD 2.69). An Analysis of Variance (ANOVA) was used to compare the outcome of interest (i.e., mean number of stars among selected products) between the three randomly assigned groups.

Tests of equivalence of conditions (based on ANOVA and chi-square analyses) indicated that random assignment was successful in generating similar groups by study condition. There were no differences between conditions in child age, BMI, race/ethnicity, or language spoken (*p* > 0.40).

Of the 90 available products, 87 were purchased by at least one participant pair. Pairs purchased an average of eight items with their USD 20 (range of 5–12 items). The most purchased products were cheese crackers (purchased by 51 pairs), shell-shaped pasta (50 pairs), and canned black beans (49 pairs). Figure 2 shows the percent of products purchased by category.

The study hypotheses received partial support. The first hypothesis that the FOP labels alone would not produce significantly different food choices compared with the no-label Control group was supported by the ANOVA results (see Table 1 and Table 2), which revealed no significant differences in terms of mean stars on chosen foods between the group with FOP labels only (2.53 stars) and the Control group (2.67 stars).

However, the second hypothesis, that the FOP + Video group would choose products that had, on average, more stars on their FOP labels relative to the FOP Only group and the no-label Control group was not entirely supported (see Table 2). Although the FOP + Video group did choose foods with significantly more stars (2.77 stars) than the FOP Only group (2.53 stars), there was no significant difference between the mean stars present on foods chosen by the FOP + Video group (2.77 stars) and the Control group (2.67 stars).

## 4. Discussion

### 4.1. Overview

The present study measured the impact of FOP nutrition labels on parent/child food choices in a controlled setting where access to FOP labels and videos explaining the labels was randomly assigned. It was hypothesized that unexplained interpretive nutrient-specific summary indicator FOP labels would not generate significantly healthier food choices than would be made by parent/child pairs without access to these labels. This hypothesis was supported, suggesting that unexplained FOP labels may not be effective in guiding consumers toward healthier food choices.

It was further hypothesized that explaining the presence and meaning of FOP labels on food packages would lead to healthier food choices, but this hypothesis was not supported. Although participants who saw the brief public-service-announcement-style video explaining the FOP labels did make healthier food choices, on average, than those who saw the labels without viewing the video, they did not make healthier food choices than those participants in the Control group (who saw neither FOP labels nor a video about the labels).

Therefore, consistent with previous research indicating that some types of nutrition labels do not affect consumers’ food choices [39], the present research suggests that the interpretive nutrient-specific summary indicator FOP labels tested here may not increase healthy food choices among certain consumers (in this case, pairs of one parent and one 5–10-year-old child), whether these labels are explained or unexplained. This matches meta-analytic findings of the effects of nutrition labeling, which find that, on average, consumers can use nutrition labels to identify healthier foods, but that labeling does not necessarily increase the consumption of these healthier options [39,40]. Similar to the present results, previous research on the combination of FOP labeling and supplementary educational materials demonstrated benefits for providing educational materials along with novel FOP labels [33]. The stronger results in the previous study may be attributable to the fact that the educational materials in that study were provided at the point of purchase, rather than beforehand as was the case with the videos in the present study.

There are many factors that influence food-related decision making (e.g., Symmank et al. [41]), and it has long been known that healthfulness is not consumers’ top priority when making food choices, falling behind factors such as expected taste, cost, and convenience [42,43]. However, concerns about nutrition do factor into food choices for many consumers [44]; therefore, providing nutrition information in a way that it is likely to be seen and understood is a worthwhile goal. An aim of this study was to investigate a FOP label type that could be understood by children, and although labels using easily decoded symbols and meaningful color-coding offer potential pathways to increase children’s interest in/awareness of healthier food options, there are other routes to attracting children’s attention to healthy options. Previous research has found that using child-friendly licensed media characters is one route to increasing child attention and preference for healthy options [45,46] and another route includes sensory activities such as exposure to healthy foods through touching and smelling that can increase child interest in eating these foods [47].

Front-of-package nutrition labeling remains a policy priority in the United States, and there is evidence from other countries that nationally mandated labels can have beneficial effects on public health nutrition (e.g., [48,49,50,51]). However, the evidence from this study suggests that the labels tested here may not be optimal for increasing healthfulness of products selected by parents and children choosing foods together. It is possible that incorporating additional colors into the tested format could be beneficial, as a recent review reports that “colour-coded labels perform relatively better than monochrome labels” in terms of producing healthier purchases [50]. Furthermore, it may be beneficial to combine information-provision approaches like the PSA-style ads used here with point-of-purchase information (e.g., in grocery aisles), as in previous research [33].

It is also important to keep in mind that the consumer/demand side of the consumption equation is not the full picture of the effects that FOP labels could have on foods and beverages consumed. The producer/supply side of the equation is also important, and potentially has a higher impact as changes made in the food supply in response to FOP labels (i.e., food manufacturers reformulating products to have less sodium or sugar in order for their FOP labels to have more stars, fewer red lights, etc.) would benefit consumer health without requiring each individual consumer to opt-in to healthier choices. Research has demonstrated that this supply-side product reformulation has indeed been the result of FOP labeling interventions (e.g., [52]). A recent meta-analysis found that labeling led the food industry to reduce the amount of sodium in their labeled products by 9% and trans fat by 64% ([53]).

### 4.2. Strengths

This study utilized a randomized controlled trial design to experimentally test the effects of FOP labels when unexplained compared with when an explanatory video supplemented the labels, and compared both of these groups with a Control group that had access to neither labels nor the video, providing a test of the most likely scenarios when FOP labeling is introduced to a given market. This study balanced the control of a laboratory experiment with the ecological validity of choosing foods and beverages from a grocery/convenience store aisle by setting up aisles in a laboratory and equipping participants with a grocery basket as if they were shopping at a local store. By asking parents and children to shop together, ecological validity was further enhanced, nearly approaching the typical experience of food shopping experienced by individuals with children. Finally, participants knew in advance of selecting products that they would keep the chosen foods and beverages, enhancing the likelihood that they would choose products they preferred.

### 4.3. Limitations

The study had limitations that should be considered. The realism of the grocery selection task was limited in several ways. Although the laboratory setting was designed to look similar to an aisle in a small grocery/convenience store, the setting was nevertheless a laboratory, and therefore not entirely naturalistic. Additionally, participants were constrained to choose a combination of foods that cost USD 20 or less, a constraint that may or may not accurately reflect participants’ regular grocery shopping occasions. It is possible that participants deduced, but did not report, the connection between the two studies. This understanding of the link between the video shown in Study 1 and the food selection task in Study 2 could have led participants to behave differently than they would have if this connection between the studies was undiscovered. Additionally, although the sample size of 175 parent/child pairs was comparable to other research examining the effects on food selection of FOP labels and educational materials (e.g., [33]), it is possible that small effects may have gone undetected due to this sample size.

### 4.4. Future Studies

Future studies could test whether repeated exposure to video messages like the one tested here would be more likely than a single exposure to lead to healthier food choices. Different modalities of conveying information about FOP labels could also be tested. In previous research [33], in-aisle informational signage was found to more effectively impact consumers to use FOP labels when making food choices. It is possible that other forms of more proximal information provision (e.g., FOP-related information provided in grocery shopping-list apps) might also lead to greater use of FOP labels and to healthier food choices. As noted above, FOP labeling systems suffer from the limitation of focusing on individual products, rather than the overall healthfulness of the diet. Future research examining point-of-purchase approaches for assembling an overall healthy diet would be worth exploring (e.g., smartphone apps that tally up nutrients across products placed into a shopping cart, calculating overall diet-level scores, rather than relying only on product-level metrics of healthfulness).

## 5. Conclusions

Unexplained FOP nutrition labels do not appear to foster healthier food choices among parent/child consumer pairs. In addition, a brief public-service-announcement-style video description of the FOP labels may also be insufficient to prompt use of the labels to make healthier food choices. It is possible that more explanation or more frequent exposure to explanatory information is necessary for FOP labels to be used to prompt healthier food choices; alternatively, it is possible that this type of FOP nutrition labeling may not significantly affect food choices among some consumers.

## Figures and Tables

**Table 1:** ANOVA of Study Condition ^a^ Predicting Mean Number of Stars on Chosen Products.

	Sum of Squares	df	Mean Square	F	*p*	η^2^
Study Condition	1.664	2	0.832	4.572	0.012	0.050
Residuals	31.295	172	0.182			

^a^ Study condition refers to the three randomly assigned groups: (1) participants who had access to star-based front-of-package (FOP) nutrition labels (products received between 1 and 4 stars based on their levels of added sugars, sodium, and saturated/trans fats) AND a public service announcement (PSA)-style television advertisement explaining the FOP labels; (2) participants who had access to the FOP labels, but not the PSA; and (3) participants who had neither FOP labels nor the PSA.

**Table 2:** Mean Difference in Number of Stars on Chosen Products by Study Condition ^a^.

		Mean Difference	SE	*t*	Cohen’s *d*	*p*_tukey_
No PSA, No FOP (*n* = 58)	vs. FOP	0.141	0.079	1.775	0.330	0.181
No PSA, No FOP (*n* = 58)	vs. PSA FOP	−0.097	0.079	−1.226	−0.227	0.439
FOP (*n* = 59)	vs. PSA FOP	−0.237	0.079	−3.008	−0.556	0.008 *

* *p* < 0.01. Note: *p*-value adjusted for comparing a family of 3. ^a^ Study condition refers to the three randomly assigned groups: (1) participants who had access to star-based front-of-package (FOP) nutrition labels (products received between 1 and 4 stars based on their levels of added sugars, sodium, and saturated/trans fats) AND a public service announcement (PSA)-style television advertisement explaining the FOP labels (labeled “PSA FOP”); (2) participants who had access to the FOP labels, but not the PSA (labeled “FOP”); and (3) participants who had neither FOP labels nor the PSA (labeled “No PSA, No FOP”).

## Data Availability

The datasets generated and/or analyzed during the current study are available in the Open Science Framework repository, https://osf.io/pcja7/ (accessed on 21 June 2023).
